# In vivo Adenoviral Gene Transfer of CGRP and Related Peptides to the Rat and Mouse

**DOI:** 10.1100/tsw.2001.404

**Published:** 2001-11-30

**Authors:** Trinity Bivalacqua, Donald Heistad, Philip Kadowitz, David Kass, Hunter Champion

**Affiliations:** Trinity Bivalacqua, Donald Heistad, Philip Kadowitz, David Kass, and Hunter Champion Division of Cardiology, Department of Medicine, The Johns Hopkins Hospital, Baltimore, MD, USA

## Abstract

Calcitonin gene-related peptide (CGRP) and adrenomedullin (ADM) play an important role in maintaining low pulmonary vascular resistance (PVR) and may be involved in modulating the pulmonary vascular response to chronic hypoxia. Adenoviral vectors encoding prepro-CGRP (AdRSVCGRP) and ADM (AdRSVADM) were used to examine the effects of in vivo gene transfer of the peptides to the lung on increases in PVR, right ventricular mass, and pulmonary vascular remodelling that occurs in chronic hypoxia in the mouse. Intratracheal administration of AdRSVCGRP or AdRSVADM, followed by 16 days of chronic hypoxia (FIO2 0.10), increased lung CGRP and ADM levels, respectively. The increase in pulmonary arterial pressure (PAP), PVR, right ventricular mass, and pulmonary vascular remodeling in response to chronic hypoxia was attenuated in animals overexpressing prepro-CGRP or ADM whereas systemic arterial pressure was not altered. In chronically hypoxic mice, increases in PAP in response to i.v. injections of angiotensin II and endothelin-1 were attenuated following in vivo gene transfer. These data show that in vivo transfer of the CGRP or ADM gene to the lung attenuates the increase in PVR and right ventricular mass, pulmonary vascular remodeling, and responsiveness in chronically hypoxic mice with little effect on the systemic circulation. In order to study the roles of RAMP 1, 2, and 3 in mediating responses to CGRP and related peptides in the pulmonary and systemic vascular beds, gene transfer of adenovirus vectors encoding CRLR and RAMP 1, 2, or 3 were delivered to the lung and/or hindlimb of the mouse. Transfection of CRLR and RAMP 2 to the pulmonary and hindlimb vascular beds of the mouse resulted in enhanced vasodilator responses to ADM, but not CGRP.

